# Spectrophotometric quantification of paracetamol and tramadol hydrochloride by chemometric calibration methods

**DOI:** 10.55730/1300-0527.3566

**Published:** 2023-05-17

**Authors:** Faysal SELİMOĞLU, Nermin PINARCIK

**Affiliations:** Department of Biotechnology, Faculty of Science, Necmettin Erbakan University, Konya, Turkey

**Keywords:** Paracetamol, tramadol hydrochloride, UV-spectrophotometry, chemometric calibration

## Abstract

The results of UV spectrophotometric analysis were analysed using partial least squares (PLS) and principal component regression (PCR) techniques to allow simultaneous evaluation of tramadol hydrochloride (TRA) and p-acetaminophen (PAR) in tablets. A calibration set of 16 mixtures, each containing PAR and TRA in various amounts, was created using a 2^4^-full fractional design. The absorbance data set for the calibration set were obtained between 215–280 nm (Δλ = 0.1 nm). Subsequently, the concentration and absorbance sets were used to generate PCR and PLS calibrations. The ratio spectra- first derivative method was devised as a solution to the same problem to compare the outcomes of the chemometric methods used for the same experiment. After the proposed methods were shown to be accurate in the testing of validation samples, they were used in analysing commercial tablet samples. The analytical results showed that PCR and PLS methods can be used as alternative methods to high-performance liquid chromatography (HPLC). Correlation coefficients were determined for the working concentration range of 6–36 g.mL^−1^ for PAR and 4–22 g.mL^−1^ for TRA. The limits of detection and quantification were calculated as 0.9104 μg.mL^−1^ and 3.0347 μg.mL^−1^, respectively. The test results of the chemometric analyses and ratio spectra- first derivative method of the commercial tablet form are in agreement with the results of the one-way ANOVA with a confidence interval of 95%. This study shows that the ratio spectra- first derivative method, PCR and PLS models based on spectrophotometric measurements are very useful and straightforward techniques for the quantitative resolution of a two-component pharmaceutical preparation, requiring little sample preparation and little time for analysis.

## 1. Introduction

**Figure 1:**
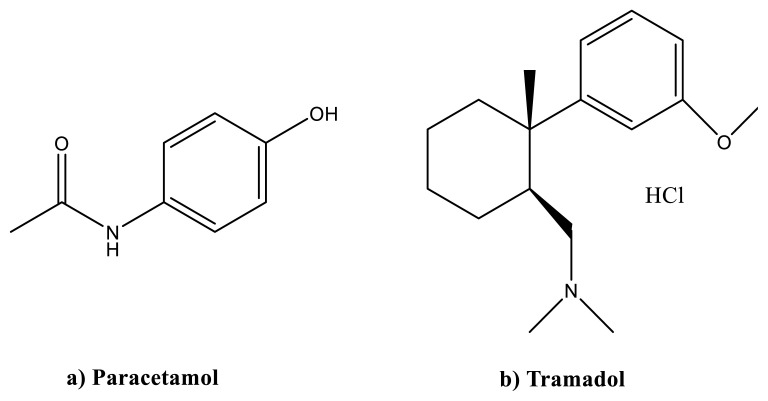
Chemical structures of **a)** paracetamol, and **b)** tramadol hydrochloride.

Paracetamol (acetaminophen) PAR is an active pharmaceutical compound that is frequently used in drug formulations alone or in combination with other active compounds. Paracetamol has analgesic and antipyretic effects on the body by reducing the intensity of pain signals to the brain and regulating body temperature in the brain [1]. It is rapidly absorbed by the gastrointestinal tract when taken orally. It is a pain killer and is used for the treatment of different types of pain as headaches migraine, menstrual pain, toothache, cold and flu infections, neuralgia, etc. It does this by reducing the formation of prostaglandins in the central nervous system and, to a lesser extent, by inhibiting the pain response in the peripheral nervous system. It reaches its highest plasma concentration within 0.5–2 h after oral administration. It quickly distributes evenly to all tissues [2]. Paracetamol’s chemical structure is depicted in Figure 1a.

Tramadol hydrochloride (TRA) is an opioid agent used for the treatment of acute and chronic pain [3]. TRA is indicated in Figure 1b. The monoaminergic effect of tramadol is similar to antidepressant drugs [4]. Tramadol hydrochloride has been widely used alone in pharmaceutical formulations or with other active compounds. To relieve symptoms of moderate to severe pain, a combination of TRA and PAR is often used [5, 6]. The combination of PAR and TRA is a pain reliever preparation that acts on the central nervous system [7]. In this respect, the quantitative analysis of a two-component mixture containing PAR and TRA in commercially available tablets is an extremely important work not only for analytical chemistry but also for the pharmaceutical industry, without the need for a prior separation step. In drug development and research studies, the quantitative resolution of bi and multicomponent mixtures may require the development of new, inexpensive, fast, and reliable analytical methods. In analytical chemistry and related fields, the direct spectrophotometric analysis of combined pharmaceutical formulations may not be possible by direct spectrophotometric measurements due to overlapping spectra of analysed substances [8].

For this reason, HPLC has been used as a standard analytical procedure to overcome the above problems. However, the HPLC procedure may not always provide acceptable separation of analytes and their correct analysis results due to unsuitable chromatographic conditions [9]. Additionally, the HPLC method is expensive and requires a long period to find optimal separation conditions for the chromatographic analysis of complex mixtures [10].

In the literature, many quantitative analytical methods have been investigated in order to the separate determination of tramadol and paracetamol in the drug industry. PAR in samples was analysed by several analytical methods including UV-spectrophotometry [11,12], HPLC [13–16], gas chromatography-tandem mass spectrometry [17], electrochemistry [18–19], capillary electrophoresis [20] and Fourier transform infrared spectroscopy (FTIR) [21,22]. The presence of tramadol can be determined in a variety of pharmaceutical products and biological materials through the use of certain analytical techniques, the use of which has been thoroughly researched and documented using UV-spectrophotometry [23–25], fluorescence spectroscopy [26], liquid chromatography-tandem mass spectrometry [27] gas chromatography-mass spectrometry (GC-MS) [28], capillary electrophoresis with electrochemiluminescence (CE-CL) [29], FT-Raman [30], voltammetry [31], can be found in the literature. Simultaneous quantitative analysis of tramadol, paracetamol, and their combinations were performed by using reverse-phase high-performance liquid chromatography [32, 33], ultra-high-performance liquid chromatography (U-HPLC) [34], UV-spectrophotometry [35,36], spectrophotometric and spectrofluorometric methods [37], normal phase liquid chromatography-tandem mass spectrometry [38,39] and FTIR [40] methods have been studied and reported.

In this study, simultaneous quantitative analysis of a binary mixture containing the drugs PAR and TRA was performed for the first time. This was achieved by performing PCR and PLS calibrations based solely on the full UV spectral measurements rather than an additional step of instrumental analysis. In the literature, the PLS calibration was applied to the analysis of the mixture PAR-TRA [41]. However, these PLS methods, based on the selection of the working wavelength with an additional procedure, are very different from our PLS application in terms of the applied experimental conditions. Another study deals with the application of second derivative spectrophotometry for quantitative estimation of related drugs in the same mixture. [42]

In order to solve the above drawbacks of classical spectrophotometric and HPLC methods [43], the use of spectrophotometric measurements combined with PCR and PLS calibration methods is very useful to solve complex pharmaceutical samples without using a separation step with low cost and short analysis time. In particular, in drug analysis, chemometric analysis methods such as PCR and PLS are widely used in quality control as well as regular testing of various complicated pharmaceutical mixtures containing two or more active ingredients without the need for a separate procedure for binary and triplicate mixtures [44–46]. Especially, the ratio spectra-derivative methods have been used as alternative methods to traditional derivative spectrophotometry to solve complex problems involving binary and ternary mixtures [47–50].

## 2. Materials and methods

### 2.1. Materials

Methanol was provided by Riedel-de Haan, and all chemicals utilized in the present study were of analytical grade (Germany). Chemicals used in the analysis: Paracetamol and tramadol hydrochloride were kindly supplied by (Abdi İbrahim Pharmaceuticals Industry and Trade. Inc %98, İstanbul-Turkey), and methanol (%99.7-Germany).

Tablets containing 325 mg of paracetamol PAR and 37.5 mg tramadol TAR were purchased from a local pharmacy and investigated in this study. These tablets were produced by Abdi İbrahim Pharmaceuticals Industry and Trade. Inc İstanbul-Turkey (Zaldiar® tablets Batch no:001825/10.2023).

### 2.2. Standard solutions

**Figure 2:**
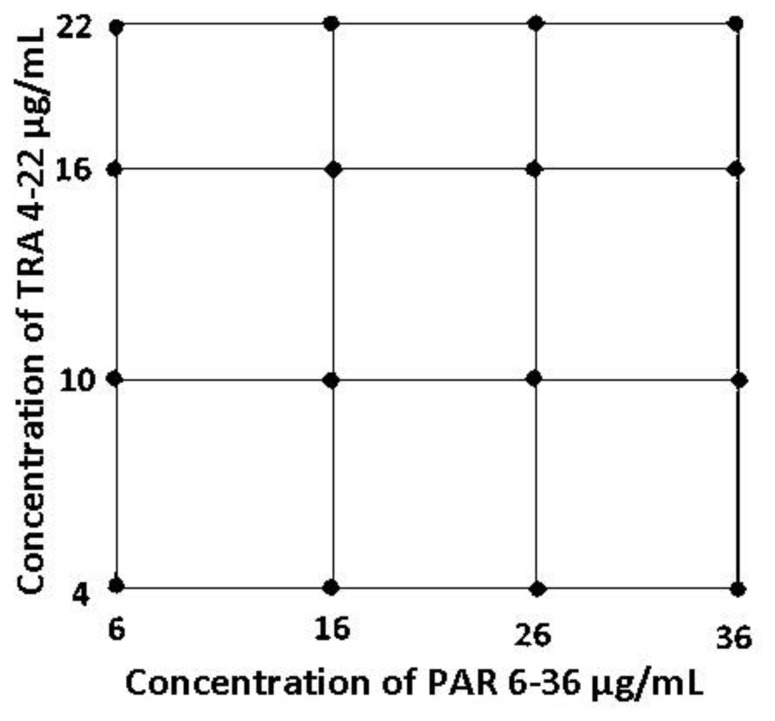
Construction of a sample set for the calibration of PCR and PLS.

Both PAR and TRA were produced as stock solutions in methanol at concentrations of 20 mg.mL^−1^ and 10 mg.mL^−1^, respectively. The solutions of the calibration and validation sets in 10 mL^−1^ calibration bottles were prepared from the above stock solutions, and then the volume of the solutions was made up to the mark (10 mL) with methanol. As shown in Figure 2, 16 different calibration solutions were generated for calibration of the PCR (6–36 μg.mL^−1^) as well as the PCR (4–22 μg.mL^−1^) itself using the standards PAR and TRA in a linear concentration range. To perform the method of the ratio spectra-first derivative method, the calibration solutions of the standards PAR and TRA were prepared separately in the concentration range of 6–36 μg.mL^−1^ and TRA 4–22 μg.mL^−1^, respectively. To test the validity of the procedures, a second validation set of the synthetic combination was used.

In the analysis of these drugs in a binary mixture and tablets, the PCR and PLS calibration models were constructed using a 2^4^-full factorial design of concentration (or calibration) set, which is a symmetric concentration. In order to control the detection results of PAR and TRA combinations by the PCR and PLS chemometric methods, a new spectrophotometric method (Ratio Spectra First Derivative Spectrophotometry) was the first suggested use of this technique to solve the problem of binary mixture analysis.

The validation of the proposed PCR and PLS calibration models and ratio spectra-first derivative method was carried out by analysing the validation samples including the test samples and standard addition samples. As a result, commercial tablets were analysed using established PCR and PLS techniques with little sample preparation, low cost, and rapid analysis time.

### 2.3. Preparation of sample solution

A mortar was used to grind ten tablets after weighing them accurately. The sample dissolved in 100 mL of methanol contained PAR and TRA and accounted for one-eighth of the total contents of the tablet. After stirring with a magnetic stirrer for one h, the tablet solution was filtered using a 0.45 m syringe filter. After that, the resulting solutions were diluted with methanol to a concentration range that was suitable for working with. The spectrum of the final sample was recorded between 215–280 nm (Δλ = 0.2 nm increment). This process was repeated 10 times.

### 2.4. Principal component regression: PCR

The principal component regression (PCR), one of the chemometric calibration methods, is based on obtaining orthogonal lines by decomposing the measured absorbance data set. The mathematical expression of the PCR model can be formulated as follows: The ratio (R) between the peak area of the individual drug and the adjusted drug concentration (C).


(1) C=a+b×A

Where a and b are the coefficients.


(2) B=P×q,

Where P is the matrix of eigenvectors and q is the C-loading given by


(3) $$\text{q}=\text{D}\times {\text{T}}^{\text{T}}\times {\text{A}}_{0}.$$

Here T^T^ is the transpose of the score matrix T. D is a diagonal matrix whose components are the reciprocal of the selected eigenvalues. Knowing b, a can be easily found using the formula


(4) $$\text{a}={\text{C}}_{\text{mean}}-{{\text{A}}_{\text{mean}}}^{\text{T}}\times \text{b},$$

Where A_mean_^T^ is the transpose of the matrix with the entries of the mean absorbance values and C_mean_ is the mean concentration of the calibration set.

#### 2.4.1. Partial least-squares: PLS

The partial least squares (PLS) method for calibration was obtained by combining the concentration and absorbance matrix as latent variables, namely A = T **×** P^T^+ E and


(5) C=UQ+F

By applying the linear regression


(6) C=a+bA

where the vector b is represented by


(7) $$\text{b}=\text{W}{\left({\text{P}}^{\text{T}}\text{W}\right)}^{-1}\text{Q}$$

the constant


(8) $$\text{a}={\text{C}}_{\text{mean}}-{{\text{A}}^{\text{T}}}_{\text{mean}}\text{b}$$

was obtained. This equation allows calculating the concentration of the unidentified active component compound in the samples.

### 2.5. Instrumentation and chemometric software

Spectral recordings were carried out by a UV-Vis spectrophotometer (UV-2550, Shimadzu). Recorded absorption data were transferred to the Microsoft Excel program and then processed by PCR and PLS algorithms in MATLAB (MathWorks Inc., USA) program. All the figures were plotted by using MATLAB software. Applications of PCR and PLS algorithms and derivative calculations of the ratio spectra were performed by using a written special m-file program in (the PLS Toolbox 3.0 component of the MATLAB ) MATLAB 7.0. Solutions during the analysis of analytes were filtered by a 0.42 pore size syringe filter.

## 3. Results and discussion

In analytical chemistry and related fields, the development of new methods or techniques may be necessary to resolve complex mixtures inexpensively and without prior separation steps. In particular, the UV spectrophotometric method is very simple and easy to use to analyse drugs in pharmaceutical dosage forms. However, direct UV spectrophotometric measurements may not provide the expected analytical result due to overlapping signals of a binary mixture in the same wavelength range. In such a case, the chemometric methods based on the use of multivariate spectral data are an option for the analysis of complex samples. In addition, the use of derivative and ratio spectra-derivative techniques of spectral data often provides successful results for the resolution of two- or three-component mixtures.

In this study, the resolution of the binary mixtures of the drugs studied was achieved by using the mathematical tools of chemometric PCR and PLS as an alternative to the traditional analytical methods. These mathematical tools were chosen because they are simple and provide an alternative to these methods. The ratio spectra first derivative method was applied to the analysis of the commercial tablets of the corresponding drugs to compare the analytical results of the chemometric methods.

### 3.1. PCR and PLS methods

**Figure 3:**
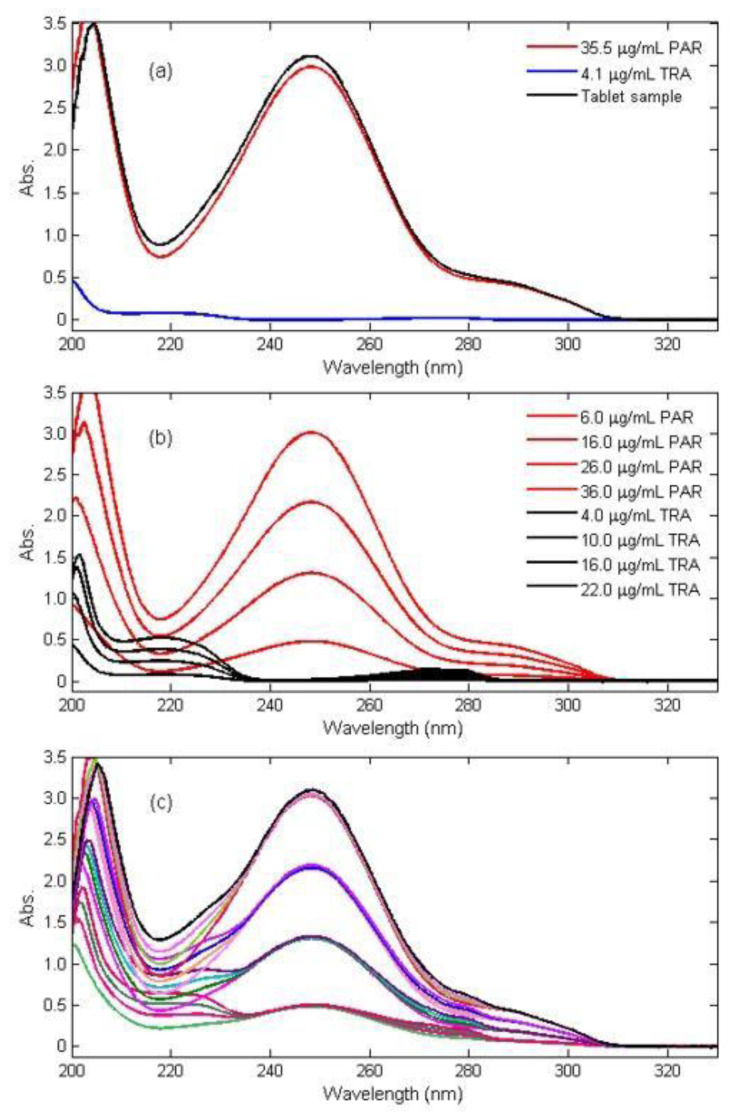
Absorption spectra of **a)** 35.5 μg mL^−1^ PAR (−), 4.1 μg mL^−1^ TAR (−) and tablet sample, **b)** 6–36 μg.mL^−1^ PAR and 4–22 μg.mL^−1^ TRA and **c)** Calibration set containing different concentration of PAR and TRA in the range of 6–36 μg.mL^−1^ and 4–22 μg.mL^−1^, respectively.

The spectra of PAR and TRA overlapped in the wavelength range of 200–330 nm, as shown in Figure 3a. Direct absorbance measurement was not an option for performing a simultaneous quantitative study of PAR and TRA in film-coated tablets under the circumstances presented here. In short, the quantification of PAR and TRA in a mixture is not possible with a simple linear regression due to the mentioned overlapping spectra of the analytes (see Figure 3a). It was concluded that this problem could be solved by applying PCR and PLS algorithms to the UV absorbance measurements to quantify both drugs in their samples. In initial experiments, the linear range of the working concentration was determined as indicated in Figure 3b.

The working concentrations range of PAR and TRA were found to be linear between 6–36 μg.mL^−1^ and 4–24 μg.mL^−1^, respectively (see Figure 3b). To generate the PCR and PLS chemometric calibrations, the calibration samples, which contained 16 different mixtures, were prepared according to the 2^4^-factorial design (see Figure 2). UV spectra of the calibration set were recorded in the spectral range of 200–330 nm as shown in Figure 3c. The composition of the calibration sample set that contains PAR and TRA in the concentration range of 6–36 μg.mL^−1^ and 4–22 μg.mL^−1^, is presented in Table 1.

A symmetric set was used to reduce random errors in the generation of the calibration set. The absorbance of the calibration set was measured in the range of 215–280 nm (Δλ = 0.1 nm). The PCR and PLS algorithms were applied to the absorbance data set and the concentration set. Without prior preprocessing of the spectral observations, the PCR and PLS algorithms were applied to the absorbance data set and used to analyse the data. The first three factors were found to be appropriate for these PCR and PLS calibration models. Concentrated PCR and PLS were utilised to measure PAR and TRA in samples.

### 3.2. Ratio spectra first derivative spectrophotometry

**Figure 4:**
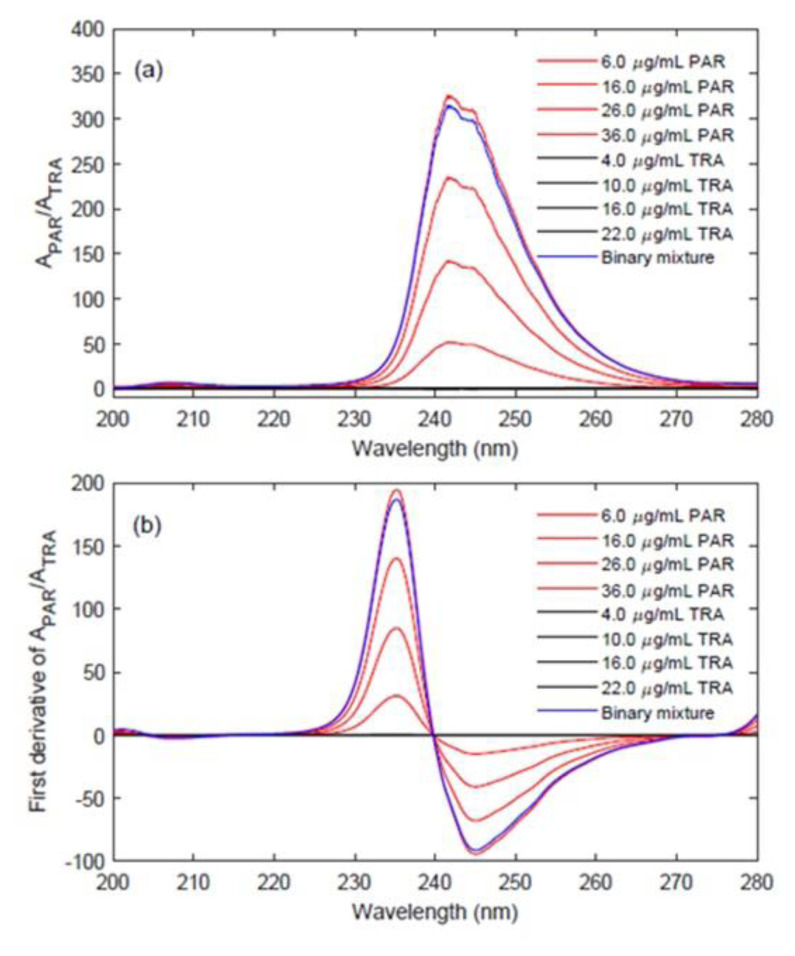
**a)** Ratio spectra of PAR (red line), TRA (black line) and their binary mixture (blue line) (when 10 μg.mL^−1^TRA was used as a divisor) and **b)** first derivative of Ratio spectra of PAR (red line), TRA (black line) and their binary mixture (blue line).

The absorption spectra of the standard solutions of PAR and TRA in the working range of 6–36μg.mL^−1^ and 4–22 μg.mL^−1^ TRA were recorded between 200–330 nm with the interval of Δλ = 0.1 nm (see Figure 3b). Similar spectral registration conditions were used for the validation and commercial tablet samples. After dividing the absorption spectra of pure PAR and its binary mixture by the reference spectrum of 10 g.mL^−1^ TRA, we were able to obtain the ratio spectra of pure PAR and its binary mixture, shown in Figure 4a. The first derivative of the ratio spectra of PAR and its binary mixture was calculated with Δλ = 4 nm in the range of 200–280 nm, as shown in Figure 4b. PAR in the samples was determined by measuring the first derived signal of the ratio spectra at 235.5 nm, which corresponds to the maximum wavelength, to obtain a calibration curve for the analysis of PAR. For the same purpose, the procedure was repeated for TRA at 280.0 nm. The statistical results for the calibration graph of the standard, PAR, and TRA are shown in Table 2.

### 3.3. Validation of the proposed methods

The validation of the chemometric calibrations (PCR and PLS techniques) and the ratio spectra first derivative method was performed using the validation parameters and the analysis of test samples and standard supplement samples. To achieve this goal, a separate validation set of nine synthetic mixtures with varying proportions of PAR and TRA was created (See Table 3). Calculations were performed using the PCR and PLS calibration methods and the first derivative of the ratio spectra method. The results of these calculations can be found in Table 3, along with the relative standard deviations and mean recoveries. Reasonable numerical values for the validation of PCR and PLS were determined in the course of the recovery investigations. With these methods, precise and accurate results were obtained for the analysis of the two compounds in the synthetic mixtures prepared in our laboratory.

To assess the extent to which the procedures were selective, a predetermined amount of the standards PAR and TRA were added to the commercial tablet solution. Solutions for the addition of the standards were prepared with three different compositions of analytes, ranging from 6–36 μg.mL^−1^ for PAR and 4–22 μg.mL^−1^ for TRA. These procedures were performed three times for every single concentration level. The results of the recoveries were collected along with their standard deviations and are shown in Table 4. When the tablets were analysed, the results showed that the excipients did not cause any interference. As a result, the presented PCR and PLS methods, in addition to ratio spectrophotometry (first derivative), were shown to be extremely suitable for the detection of PAR and TRA in tablets intended for oral ingestion.

The efficiency of chemometric calibration models is described in several ways. The most common definition of the standard error of prediction (SEP) and the standard error of calibration is denoted by (SEC).


(9) $$SEP/SEC=\sqrt{\frac{\sum_{i=1}^{n}\left(C_{i}^{Added}-C_{i}^{Found}\right)}{n}}$$

Where ${\text{C}}_{i}^{Added}$ shows the added concentration of the drug; ${\text{C}}_{i}^{Found}$ is the predicted concentration of the drug and n is the total number of synthetic mixtures.

The calculation used to determine the prediction of residual errors (PRESS) of the calibration phase was as follows:


(10) $$PRESS=\sum_{i=1}^{n}{\left(C_{i}^{Added}-C_{i}^{Found}\right)}^{2}$$

In the implementation of PCR and PLS chemometric calibrations with the first three factors, SEP, SEC, and PRESS values are listed in Table 5.

The parameters of the calibration curves for PAR and TRA are presented in Table 2. Higher correlation coefficients were found for the working concentration range of 6–36 g.mL^−1^ for PAR and 4–22 g.mL^−1^ for TRA, which can be seen in this table. The numerical results of the LOD and LOQ values are given in Table 2. The results showed that the validity of the methods was within acceptable limits in terms of the applicability of the proposed procedures to the analysis of samples.

### 3.4. Analysis of commercial tablets

In the application of chemometric methods to real samples, PCR and PLS techniques and the spectrophotometric tool Ratio Spectra-First Derivative were used for quantitative analysis of PAR and TRA in commercial tablets. Table 6 shows the results of the study on various commercially available tablets. In applying the analytical procedures, the results were obtained from the average of ten replicates in the statistical analysis in the experiments with the commercial tablets. Then SD and RSS values were calculated from the analysis results given in Table 6. Both PCR and PLS calibrations and the ratio spectra method (first derivative) were found to give satisfactory results for the simultaneous quantitative analysis of PAR and TRA in commercial drug formulations. The assay results obtained by the proposed methods and the information on the label of the tablet formulation agreed quite well.

A comparison was made between the experimental recovery results obtained by applying the PCR and PLS techniques to commercially available tablets and the results obtained by the recently proposed ratio spectra first derivative method. To compare the analytical results of the commercial tablets obtained by the chemometric method and ratio spectra- first derivative methods, the one-way test ANOVA with a confidence limit of 95% was applied. Statistical analysis showed that the assay results obtained with the proposed methods did not differ significantly from each other in any way (see Table 7).

## 4. Conclusions

In this article, for the first time, two chemometric methods (PCR and PLS) based on the complete UV absorbance measurements were proposed as alternative methods for quantitative resolution of two-component mixtures and commercial tablets of the studied drugs (PAR and TRA) with low cost and short analysis time and without preseparation step. In order to verify the results of chemometric analysis, the newly proposed ratio spectra- first derivative method was used for the same problem of this study. The test results of ANOVA show that the proposed chemometric methods and the method of the first derivative of the ratio spectrum give comparable results in the quantitative analysis of the commercial tablets. We concluded that the newly proposed ratio spectra-first derivative is an alternative method to second derivative spectrophotometry for solving the same problem due to its simplicity and applicability. As a result, the newly proposed PCR and PLS calibrations and ratio spectra first derivative were useful chemometric methods for quality control and routine analysis of PAR and TRA in commercial tablets.

## Figures and Tables

**Table 1:** Concentration set containing PAR and TRA prepared by using 2^4^ factorial design.

	Concentration (μg.mL^−1^)	
C	PAR	TRA
1	6.00	4.00
2	6.00	10.00
3	6.00	16.00
4	6.00	22.00
5	16.00	4.00
6	16.00	10.00
7	16.00	16.00
8	16.00	22.00
9	26.00	4.00
10	26.00	10.00
11	26.00	16.00
12	26.00	22.00
13	36.00	4.00
14	36.00	10.00
15	36.00	16.00
16	36.00	22.00

The chemometric calibrations and their corresponding spectra are presented in Figure 3c.

**Table 2:** Results of the statistical analysis of the calibration curves.

Wavelength	235.5 nm	280.0 nm
m	0.0168	5.4349
n	0.0098	−2.0971
r	0.9996	1.0000
SE(m)	0.0003	0.0215
SE(n)	0.0051	0.5112
SE(r)	0.0047	0.4805
LOD (μg mL^−1^)	0.9104	0.2822
LOQ (μg mL^−1^)	3.0347	0.9406

**LOD :** Limit of detection

**LOQ :** Limit of quantitation

**Table 3:** The results of recovering PAR and TRA from a binary mixture using PLS and PCR techniques.

		Recovery (%)					
Added (μg mL^−1^)		PCR		PLS		RS-FDS	
PAR	TRA	PAR	TRA	PAR	TRA	PAR	TRA
6.0	4.0	100.0	99.5	100.8	101.4	101.7	101.0
16.0	4.0	99.8	102.9	100.2	99.6	99.7	100.5
26.0	4.0	97.6	102.8	98.4	104.1	97.1	100.2
36.0	4.0	101.0	104.4	100.7	100.5	101.5	98.5
35.0	4.0	100.8	101.0	101.4	99.2	101.3	101.1
35.0	10.0	98.5	100.2	99.0	98.7	99.3	98.5
35.0	16.0	98.9	98.7	99.3	99.3	99.8	96.8
35.0	22.0	98.0	100.9	98.5	99.7	98.8	99.1
35.5	4.1	99.3	101.3	99.7	98.8	99.7	101.1
	Mean	99.3	101.3	99.8	100.1	99.9	99.6
	SD	1.19	1.80	1.07	1.71	1.46	1.53
	RSD	1.19	1.78	1.07	1.71	1.46	1.53

**SD**: Standard deviation

**RSD**: Relative standard deviation

**Table 4:** Recoveries obtained by the conventional addition method using the proposed analytical methods.

	Added (μg mL^−1^)		Found (μg mL^−1^)					
			PCR		PLS		RS-FDS	
	PAR	TRA	PAR	TRA	PAR	TRA	PAR	TRA
Formulation	2.0	3.5	2.02	3.58	2.05	3.55	2.07	3.48
Formulation	12.0	8.5	12.16	8.73	12.16	8.69	12.23	8.20
Formulation	22.0	18.5	22.46	19.05	22.53	19.10	22.88	18.04
			Recovery (%)					
			PCR		PLS		RS-FDS	
			PAR	TRA	PAR	TRA	PAR	TRA
			101.1	102.4	102.5	101.3	103.3	99.4
			101.3	102.7	101.4	102.3	101.9	96.5
			102.1	103.0	102.4	103.2	104.0	97.5
			RSD (%)					
			PCR		PLS			
			PAR	TRA	PAR	TRA	PAR	TRA
			3.25	1.89	1.38	3.19	0.91	3.42
			2.59	1.01	2.63	0.94	2.16	0.64
			0.71	0.57	0.75	1.19	0.44	1.46

* Data were compiled from the average of three separate experiments conducted at each concentration level.

**Table 5:** Statistical parameters for PCR and PLS methods.

Parameters	PCR		PLS	
	PAR	TRA	PAR	TRA
SEC	0.7030	0.1473	0.3730	0.5160
SEP	0.3013	0.1429	0.2173	0.1783
PRESS	0.4125	0.9391	0.3480	0.7949

**SEC**: Standard error of calibration

**SEP**: Standard error of prediction

**PRESS:** Prediction error sum of squares.

**Table 6:** Determination results of PAR and TRA in tablets by PCR and PLS.

	mg/tablet					
	PCR		PLS		RS-FDS	
Exp No.	PAR	TRA	PAR	TRA	PAR	TRA
1	320.6	38.9	321.1	37.2	329.0	37.5
2	329.4	36.8	328.9	37.7	325.3	37.4
3	327.3	37.7	327.5	37.5	326.6	38.3
4	323.7	38.1	324.8	37.1	320.4	37.8
5	331.8	38.9	329.3	36.8	328.5	37.5
6	328.3	37.9	328.6	36.4	325.3	36.6
7	329.0	38.6	329.5	38.4	325.3	36.0
8	330.0	36.9	328.8	37.4	327.1	37.4
9	329.2	36.6	329.1	35.6	326.3	38.6
10	328.2	37.1	327.4	38.1	329.1	39.2
Mean:	327.7	37.8	327.5	37.2	326.3	37.6
SD:	3.27	0.87	2.65	0.82	2.55	0.92
RSD:	1.00	2.30	0.81	2.20	0.78	2.46

**SD:** Standard deviation

**RSD:** Relative standard deviation

**Table 7:** One-way ANOVA test for the determination results obtained by applying PCR, PLS, and Rs-FDS to commercial tablets.

	Source of variation	SS	df	MS	F	P-value	F crit
PAR	Between groups	13.4	2	6.68	0.92	0.41	3.32
	Within groups	217.8	30	7.26			
	Total	231.2	32				
TRA	Between groups	1.9	2	0.93	1.36	0.27	3.32
	Within groups	20.5	30	0.68			
	Total	22.4	32				

**SS:** Sum of squares

**Df:** Degree of freedom

**MS**: Mean square
